# Contextual Social Cognition Impairments in Schizophrenia and Bipolar Disorder

**DOI:** 10.1371/journal.pone.0057664

**Published:** 2013-03-08

**Authors:** Sandra Baez, Eduar Herrera, Lilian Villarin, Donna Theil, María Luz Gonzalez-Gadea, Pedro Gomez, Marcela Mosquera, David Huepe, Sergio Strejilevich, Nora Silvana Vigliecca, Franziska Matthäus, Jean Decety, Facundo Manes, Agustín M. Ibañez

**Affiliations:** 1 Institute of Cognitive Neurology (INECO) & Institute of Neuroscience, Favaloro University, Buenos Aires, Argentina; 2 National Scientific and Technical Research Council (CONICET), Buenos Aires, Argentina; 3 Universidad Autónoma del Caribe, Barranquilla, Colombia; 4 Pontifical Catholic University of Argentina, Buenos Aires, Argentina; 5 Laboratory of Cognitive and Social Neuroscience, Universidad Diego Portales, Santiago, Chile; 6 Interdisciplinary Center for Scientific Computing, University of Heidelberg, Heidelberg, Germany; 7 Departments of Psychology and Psychiatry, and Center for Cognitive and Social Neuroscience, University of Chicago, Chicago, Illinois, United States of America; 8 CARI University Hospital, Barranquilla, Colombia; 9 Resurgir Psychiatric Clinic, Barranquilla, Colombia; 10 University of Cologne, Cologne, Germany; 11 Instituto de Humanidades (IDH) de la Facultad de Filosofía y Humanidades, Universidad Nacional de Córdoba, Córdoba, Argentina; University of Buenos Aires, Argentina

## Abstract

**Background:**

The ability to integrate contextual information with social cues to generate social meaning is a key aspect of social cognition. It is widely accepted that patients with schizophrenia and bipolar disorders have deficits in social cognition; however, previous studies on these disorders did not use tasks that replicate everyday situations.

**Methodology/Principal Findings:**

This study evaluates the performance of patients with schizophrenia and bipolar disorders on social cognition tasks (emotional processing, empathy, and social norms knowledge) that incorporate different levels of contextual dependence and involvement of real-life scenarios. Furthermore, we explored the association between social cognition measures, clinical symptoms and executive functions. Using a logistic regression analysis, we explored whether the involvement of more basic skills in emotional processing predicted performance on empathy tasks. The results showed that both patient groups exhibited deficits in social cognition tasks with greater context sensitivity and involvement of real-life scenarios. These deficits were more severe in schizophrenic than in bipolar patients. Patients did not differ from controls in tasks involving explicit knowledge. Moreover, schizophrenic patients’ depression levels were negatively correlated with performance on empathy tasks.

**Conclusions/Significance:**

Overall performance on emotion recognition predicted performance on intentionality attribution during the more ambiguous situations of the empathy task. These results suggest that social cognition deficits could be related to a general impairment in the capacity to implicitly integrate contextual cues. Important implications for the assessment and treatment of individuals with schizophrenia and bipolar disorders, as well as for neurocognitive models of these pathologies are discussed.

## Introduction

Social cognition refers to the processes that subserve behavior in response to co-specifics [1] and enable individuals to take advantage of being part of a social group [2]. The construct of social cognition involves several domains including emotional processing, empathy and social norms knowledge, among others. Numerous studies [3]–[10] have reported social cognition deficits in people who suffer from neuropsychiatric conditions [9], [10] including patients with schizophrenia (SC) [11], [12] and bipolar disorders (BD) [13], [14].

### Affective and Social Cognition Deficits in SC and BD

Emotional processing is an important topic of research in SC and BD. In patients with SC, several studies have evidenced a deeper impairment affecting basic emotions expression [15], [16], facial emotion recognition [4], [5] and the identification of emotions from human shapes and body motion [17]. Electrophysiological [18], [19] and neuroimaging studies [20], [21] have reported reduced amplitude (N170, a face specific component) and decreased activation (fusiform gyrus and amygdala) during responses to facial emotional stimuli. On the other hand, abnormalities in emotion recognition have also been observed in BD I [7], [22], [23] and BD II patients [24]. Functional magnetic resonance imaging studies have reported that depressed and manic patients tended to overactivate brain regions in response to happy and sad faces, respectively, and both showed an increased neural response to fear faces [25]. A recent meta-analytic review found that emotional processing could be considered a trait marker of BD [26]. In contrast, studies comparing both disorders demonstrated that SC patients exhibit greater deficits in emotional processing than BD patients [27], [28].

Deficits in empathy have also been reported in SC and BD patients. Reduced levels of empathy, measured by self-report questionnaires, have been described in both SC [3], [29]–[31] and BD I and II patients [6], [32]. Moreover, experimental designs measuring empathy have evidenced impaired emotional perspective taking in SC patients [33]. In BD I and II patients, emphatic emotional perspective taking is negatively influenced by residual manic and depressive symptoms [8]. No comparison among groups has been performed.

In addition, SC patients present impairments in the ability to understand social knowledge about their culture [34], [35]. They also fail to discriminate among inappropriate behaviors. However, they are able to identify violations of social norms [36]. Although most of BD I patients cannot accurately judge social interactions, [37] they exhibit appropriate social norms knowledge [38]. No studies have assessed social norms knowledge in BD II or compared SC and BD patients.

In summary, SC and BD patients exhibit deficits in several social cognition domains including emotional processing, empathy and social norms knowledge, with a tendency of higher impairments in SC patients. However, these findings were obtained using tasks that can be solved with relatively abstract and universal rules learned by explicit knowledge (e.g., explicit social norms during specific social interactions). Contrarily, other social cognition tasks require the implicit inference of contextual cues and putting social meaning into focus [39]. Context-sensitive ecological measures similar to real-life situations should be applied when studying disorders such as SC and BD. These tools can provide a more realistic approach of the social cognition profile of these pathologies [10], [40]. For example, a recent study [41]detected executive deficits in BD euthymic patients using tasks with real-life context, a finding that could not have been observed through traditional executive tests.

### Contextual Social Cognition in SC and BD

The core of social cognition requires the integration of contextual information in order to gain access to the social meaning [1], [39], [42]. In SC patients, deficits in context processing may be a core deficit that underlies cognitive and social cognition impairments [43], [44]. Individuals with SC showed deficits in using contextual cues to assess the emotional intensity of facial expressions [45]. In addition, they showed deficits in performance on social perception tasks that involve contextual cues [46]. Social context does not play such an important role for SC patients in emotion recognition during conversations [47]. In contrast, context processing has been less studied in BD. These patients may show a context processing deficit of nonsocial information, though less severe than SC patients [48]. In BD, the effects of context on social cognition performance have not been directly studied.

There is emerging evidence that SC and BD might share genetic susceptibility [49], [50]. Furthermore, these conditions have similar ages of onset, sex distributions, and prevalence [51]. Neuroimaging studies have also revealed similarities between SC and BD [52], [53]. In addition, these disorders partially share symptomatology and are often co-morbid [54]. Although a comparison of social cognition deficits in both SC and BD would better serve to understand the physiopathology of these disorders, few studies of this kind exist, and none have tested contextual social cognition.

### The Goal of this Study

This study explored the performance of SC and BD patients on emotion recognition, empathy and social norms knowledge, evaluating the possible role of context processing. We included tasks with different levels of contextual dependence and involvement of real-life scenarios: two emotion recognition tasks with low (emotional morphing) and high [The assessment of social inference test (TASIT)] context processing requirements, an ecological measure of empathy, and a questionnaire of explicit (abstract and non-context dependent) knowledge of social norms. Taking into account that social cognition deficits may be modulated by executive deficits [24] and subsyndromic symptomatology [26], we explored the association between clinical symptoms, EF and social cognition measures. Finally, given that basic emotion impairments would preclude more complex empathy deficits, we carried out a logistic regression analysis to examine whether the use of more basic skills in emotional processing could predict performance on empathy tasks. We expected that SC and BD patients would have deficits in social cognition tasks with high context processing demands, and that these impairments would be less severe in BD than in SC patients. We also hypothesized that basic impairments in emotional and executive processing would be related to social cognition deficits in both disorders.

## Materials and Methods

### Participants

Sixty subjects (SC: n = 15; BD: n = 15; healthy comparison subjects: n = 30) participated in the present study. Patients in the SC and BD groups were selected from the outpatient populations of Institute of Cognitive Neurology (INECO) according to the following inclusion criteria: 1) subjects older than 18 years old (mean = 34.46; SD = 10.03); 2) diagnosed with paranoid SC or Type-II BD according to the diagnostic and statistical manual of mental disorders (DSM-IV) criteria [55]. All patients with SC were clinically stable. The diagnoses were confirmed using the schedules of clinical assessment in neuropsychiatry (SCAN) [56]. At the time of the assessment, five (33.33%) BD patients were euthymic, four (26.66%) depressed and six (40%) in remission with subsyndromal symptoms. The euthymic state was defined by scores less than or equal to 8 points according to the Montgomery-Asberg depression rating scale (MADRS) [57] and less than or equal to 6 according to the Young mania rating scale (YMRS) [58] for at least 8 weeks. The depression episode was defined by a score 20 on the MADRS.

Exclusion criteria were 1) other axis-I diagnoses, except for generalized anxiety disorder and 2) a history of mental retardation, neurological disease, or any clinical condition that might affect cognitive performance.

Thirty healthy comparison subjects (controls) were recruited and matched one by one with each patient group. Matching criteria were sex, age (±3 years) and years of education (±3 years). Control subjects were recruited from a larger pool of volunteers who did not have a history of drug abuse or a family history of neurodegenerative or psychiatric disorders.

### Ethics

All participants provided written informed consent (as outlined in the PLoS consent form) in agreement with the Helsinki declaration. Although some of the participants have a diagnosis of BD or SC, neither of these disorders implies a reduced capacity to consent. In the case of those patients whose capacity to consent was compromised, next of kin, career takers or guardians consented. The Ethics Committee of the Institute of Cognitive Neurology approved this study. All data was analyzed anonymously.

### Clinical and Executive Functions (EF) Assessments

All participants completed a series of psychiatric questionnaires and other measures to establish a clinical symptom profile. Participants’ intellectual level was assessed using Raven’s standard progressive matrices [59]. We measured depression using the MADRS [57]. We used the YMRS to determine the degree of mania [58]. In SC patients, the degree of psychopathology was measured using the positive and negative syndrome scale (PANSS) [60].

All participants were evaluated using the INECO frontal screening (IFS) [61], which has been shown to successfully detect executive dysfunction [61], [62]. This test includes the following eight subtests: (1) motor programming (Luria series, “fist, edge, palm”); (2) conflicting instructions (subjects were asked to hit the table once when the administrator hit it twice, or to hit the table twice when the administrator hit it only once); (3) motor inhibitory control; (4) numerical working memory (backward digit span); (5) verbal working memory (months backwards); (6) spatial working memory (modified Corsi tapping test); (7) abstraction capacity (inferring the meaning of proverbs), and (8) verbal inhibitory control (modified Hayling test). The maximum possible score on the IFS is 30 points.

### Social Cognition Measures

#### Emotional morphing

Emotional morphing is a facial expression recognition task featuring six basic emotions (happiness, surprise, sadness, fear, anger and disgust) taken from pictures of affect series [63]. The pictures have been morphed for each prototype emotion and for a neutral state [64]. Facial morphing is generated by taking a variable percentage of the shape and texture differences between the two standard images 0% (neutral) and 100% (full emotion) in 5% steps (500 ms for each image). The 48 morphed facial stimuli were randomly presented on a computer screen until the patient indicated a response on the keyboard. Participants were asked to respond as soon as they recognized the facial expression, and then to identify the facial expression from a forced choice list of six options. This task measures the accuracy of emotion recognition and reaction times (RTs).

#### The Awareness of Social Inference Test (TASIT)

TASIT is a sensitive test of social perception developed for studies on neuropsychiatry and comprises videotaped vignettes of everyday social interactions [65]–[67]. We considered only part 1, called the Emotion Evaluation Test (EET), which assesses recognition of spontaneous emotional expression (fearful, surprised, sad, angry and disgusted). In the EET, speaker demeanor (voice, facial expression and gesture) together with the social situation indicate the emotional meaning. This task introduces contextual cues (e.g., prosody, facial movement, and gestures) and additional processing demands (e.g., adequate speed of information processing, selective attention, and social reasoning) which are absent when viewing static displays. The brief EET comprises a series of 20 short (15–60 seconds) videotaped vignettes of trained professional actors interacting in everyday situations. In some scenes, there is only one actor talking, who is either on the telephone or talking directly to the camera. Other scenes depict two actors and instructions are given to focus on one of them. All scripts are neutral in content and do not lend themselves to any particular emotion. After viewing each scene, the test participant is instructed to choose from a forced-choice list the emotion expressed by the focused actor.

#### Empathy for Pain Task (EPT)

The EPT evaluates empathy for pain in the context of intentional and accidental harm, as well as control situations. The task consists of the successive presentation of 24 animated situations with two persons [68]. Three kinds of situations were depicted: intentional pain in which one person (passive performer) is in a painful situation caused intentionally by another (active performer), e.g., stepping purposely on someone’s toe (pain caused by other); accidental pain where one person is in a painful situation accidentally caused by another; and control or neutral situations (e.g., one person receiving a flower given by another).

Importantly, the faces of the protagonists were not visible and there was no emotional reaction visible to the participants. We measured the ratings and RTs to *situation comprehension* (e.g., “press the button as soon as you understand the situation”). In addition, we assessed 7 questions about the following qualities: the *intentionality*, e.g., the accidental or deliberate nature of the action; the empathic concern (how sad you feel for the passive performer); the degree of *discomfort* (for the passive performer); the *harmful behavior* (how bad was the purpose of the active performer); the *valence behavior* of the active performer (how much positive emotion he/she felt in performing the action); the *correctness* of the action (moral judgment); and finally *punishment* (how much penalty this action deserves). Each question was answered using a computer-based visual analogue scale giving 7 different pain ratings by trial. Accuracy, RTs and ratings were measured.

#### Social Norms Questionnaire (SNQ)

We used the SNQ consisting of 20 yes-no questions [69]. Participants were asked to determine whether a behavior would be appropriate in the presence of an acquaintance (not a close friend or family member) based on current social norms. The SNQ calculates the break score, defined as the total number of errors made in the direction of breaking a social norm, and the over-adhere score, defined as the total number of errors made in the direction of over adherence to a perceived social norm.

### Data Analysis

The demographic, neuropsychological and experimental data were compared between groups using ANOVA and Tukey’s HSD post-hoc tests (when appropriate). The ANOVA results were also corrected for multiple comparisons using the Tukey’s test. When analyzing categorical variables (gender) chi square test were applied. To control for the influence of BD main clinical symptoms (depression and mania) on social cognition tasks, we applied an ANCOVA test adjusted for MADRS and YMRS scores. We reported only effects that were still significant after covariation. In addition, we performed Pearson’s correlations to examine the associations between the clinical scales/EF scores and the social cognition tasks. The significance of all correlations has been corrected for multiple comparisons using the Sidak method. The adjusted α level after correction for multiple correlations was set at.0008. The *α* value for all other statistical tests (not related to correlations) was set at .05.

Finally, we used a generalized linear model (GLM) to perform a nonlinear regression of the category vector (emotion scores) over a linear combination of variables (empathy). The empathy scores of the three pain situation types (neutral, accidental and intentional) were modeled as binomial variables with probability of success parameterized into two independent variables: the total score of TASIT and the accuracy of emotional morphing task. Probabilities were obtained under the logistic regression framework together with p-values significances for the proposed predictors (File S1 general logistic regression details). This procedure determines whether performance on the empathy task has a significant dependence on emotional tasks performance.

## Results

Demographic, clinical and neuropsychological results are provided in Table 1. Fourteen (93.3%) SC patients were taking atypical antipsychotics, 5 (33.3%) typical antipsychotics, 3 (20%) benzodiazepines, 2 (13.3%) mood stabilizers and 2 (13.3%) SSRI antidepressants, either alone or in combination. Regarding BD patients, 14 (93.3%) were taking mood stabilizers, 10 (66.6%) atypical antipsychotics, 4 (26.6%) SSRI antidepressants, 2 (13.3%) benzodiazepines, and 1 (6.6%) typical antipsychotics, either alone or in combination.

**Table 1:** Demographic, clinical and executive functions assessments.

		SC(n = 15)	BD(n = 15)	CTR(n = 30)	SC vs. CTR	BD vs. CTR	SC vs. BD
Demographics	Age (years)	33.0 (7.9)	35.9 (11.8)	34.3(9.3)	N.S	N.S	N.S
	Gender (F:M)	3:11	11:4	15:15	N.S	N.S	0.001
	Education (years)	9.3 (4.1)	12.2 (4.3)	11.5 (3.8)	N.S	N.S	N.S
	Raven	41.4 (3.9)	40.4 (6.0)	41.3 (6.1)	N.S	N.S	N.S
Clinical Profile	MADRS	9.46 (8.74)	11.2 (8.7)	3.3 (3.2)	0.01	0.0009	NS
	YMRS	4.06 (2.37)	5.3 (2.4)	0.5 (0.7)	0.0001	0.0001	NS
	PANSS						
	Positive	20.9 (7.4)					
	Negative	10.7 (3.6)					
	General	26.4 (12)					
	Total	58.0 (20.6)					
Executive Functions	IFS Total Score	16.53 (4.8)	21.26 (5.0)	25.86 (2.6)	0.001	0.002	0.003
	Motor series	2.4 (0.8)	2.53 (0.8)	2.9 (0.5)	N.S	N.S	0.01
	Conflicting instructions	1.6 (1.2)	2.33 (0.8)	2.86 (0.3)	0.0001	NS	0.03
	Go- no go	1.6 (1.2)	2.06 (0.7)	2.7 (0.5)	0.0004	NS	NS
	Backward digits span	2.46 (1.5)	2.73 (1.7)	4.20 (1.0)	0.005	0.03	NS
	Verbal Working memory	0.73 (0.79)	1.66 (0.7)	1.8 (0.6)	0.0001	NS	0.002
	Spatial working memory	3.13 (1.3)	3.26 (0.7)	3.6 (0.7)	NS	NS	NS
	Abstraction capacity	1.2 (0.67)	1.86 (0.9)	2.5 (0.6)	0.0001	0.02	0.04
	Verbal inhibitory control	3.4 (1.8)	4.8 (1.6)	5.43 (1.1)	0.0002	NS	0.02

MADRS: Montgomery-Asberg Depression Rating Scale; YMRS: Young Mania Rating Scale. PANSS: positive and negative syndrome scale. IFS: INECO frontal screening.

### Demographic Data

There were no significant differences in age (*F*(2,57) = .34, *p* = 0.71), education (*F*(2,57) = 2.23, *p* = .11) or IQ (F(2,57) = .16, *p* = .85) between groups. No differences in gender were observed between SC patients and controls (*X* ^2^(1) = 1.99, *p* = 0.15) or BD patients and controls (*X* ^2^(1) = 1.90, *p* = 0.16).

### Clinical and Neuropsychological Assessments

Significant differences (*F*(2,57) = 8.99, *p*<.01) between groups were observed in the MADRS total score. Post-hoc comparisons (Tukey HSD, MS = 42.62, df = 57) revealed more depressive symptoms in SC (*p*<.05) and BD patients (*p*<.01) compared to controls. In addition, between group differences (*F*(2,57) = 43.68, *p*<.01) were observed in the YMRS scores. Post-hoc analysis showed that SC (*p<.*01) and BD patients (*p<.*01) scored significantly higher than controls. Taking into account the inclusion of euthymic, depressed and subsyndromatic BD patients, we considered the scores on the YMRS and MADRS as covariables in the social cognition performance analysis.

Regarding the IFS total score, significant differences between groups were observed (F(2,57) = 28.95, *p<.*01). Post-hoc analysis (Tukey HSD, MS = 15.47, df = 57.00) showed that SC patients (*p<.*01) performed worse compared to controls. BD patients also performed worse than controls (*p<.*01), but significantly better than SC patients (*p<.*01). A detailed comparison of performance on the eight IFS subtests (File S2 executive functions assessment) indicated that SC and BD patients exhibited verbal working memory deficits. Both patient groups also performed lower than controls in the abstraction capacity, but SC patients had a lower performance than BD patients. Furthermore, SC patients showed deficits in conflictive instructions and inhibitory control.

In summary, both patient groups showed higher levels of depression and mania compared with controls. Regarding EF, both patient groups performed worse than controls. However, SC patients exhibited greater executive deficits than BD patients.

### Social Cognition Measures

**Figure 1:**
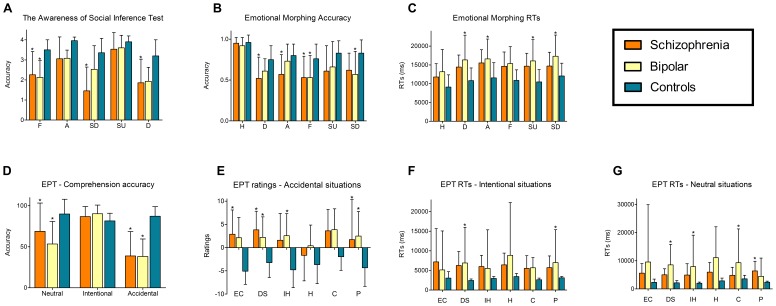
Significant differences between groups in social cognition tasks. (A) TASIT (accuracy per category). A = anger; D = disgust; SD = sadness; F = fear; SR = surprise. (B) Emotional morphing (accuracy per category). (C) Emotional morphing reaction times. H = happiness; D = disgust; A = anger; F = fear; SU = surprise; SD = sadness. (D) Empathy for pain task, comprehension accuracy. (E) Empathy for pain task, ratings for accidental situations. (F) Empathy for pain task, reaction times for intentional situations. (G) Empathy for pain task, reaction times for neutral situations. EC = empathic concern; DS = discomfort; IH = intention to hurt; H = happiness; C = correctness; P = punishment. Asterisk (*) indicates significant differences.

Figure 1 summarizes the significant differences between groups.

#### Emotional processing

Descriptive statistics and comparisons between groups are provided in Table 2. Significant differences were observed regarding accuracy on recognition of six categories of emotion (*F*(10,28) = 1.88, *p<.*05). Post-hoc analysis (Tukey HSD, MS = .04, df = 308.51) revealed a poorer accuracy performance in SC patients for emotions of disgust (*p<.*05), anger (*p<.*05) and fear (*p<.*05) than controls. Compared to controls, BD patients performed poorer on fear (*p<.*05) and sadness (*p<.*05) recognition.

**Table 2:** Emotional morphing accuracy and reaction times.

		SC(n = 15)	BD(n = 15)	CTR(n = 30)	SC vs. CTR	BD vs. CTR	SC vs. BD
Accuracy	Happiness	0.95 (0.07)	0.92 (0.1)	0.96 (0.09)	NS	NS	NS
	Disgust	0.52 (0.26)	0.61 (0.15)	0.75 (0.17)	0.03	NS	NS
	Anger	0.57 (0.24)	0.73 (0.21)	0.8 (0.14)	0.03	NS	NS
	Fear	0.53 (0.26)	0.53 (0.27)	0.76 (0.18)	0.03	0.03	NS
	Surprise	0.61 (0.31)	0.66 (0.31)	0.83 (0.15)	NS	NS	NS
	Sadness	0.62 (0.21)	0.57 (0.28)	0.82 (0.16)	NS	0.01	NS
RTs	Happiness	11769.0 (3540.2)	13164.7 (5887.2)	9087.6 (3272.4)	NS	NS	NS
	Disgust	14414.7 (3223.2)	16321.3 (6579.5)	10807.1 (3367.4)	NS	0.006	NS
	Anger	15536.0 (3477.0)	16587.6 (5290.0)	11545.6 (4025.3)	NS	0.02	NS
	Fear	14627.8 (3798.8)	15343.1 (4491.6)	10857.7 (2836.9)	NS	NS	NS
	Surprise	14635.5 (3472.8)	16065.8 (5635)	10474.9 (3290.6)	NS	0.004	NS
	Sadness	14697.0 (3675.2)	17297.3 (5635.5)	12036.6 (3402.8)	NS	0.01	NS

RTs = Reaction Times.

In addition, differences between groups (*F*(10.28) = 2.52, *p<.*01) were observed in RTs. Post-hoc analysis (Tukey HSD, MS = 4.34, df = 93.60) showed that BD patients had significantly slower RTs compared to controls for the emotions of disgust (*p<.*01), anger (*p<.*05), surprise (*p<.*01) and sadness (*p<.*05). There were no significant differences between SC and controls or SC and BD patients.

Regarding TASIT, there were significant differences between groups (*F*(10,285) = 23.82, *p<.*01) on the task performance (see Table 3). Post-hoc analysis (Tukey HSD, MS = 1.35, df = 239.90) revealed that both BD (*p<.*01) and SC (*p<.*01) patients had a lower total score than controls. However, SC (*p<.*01) performed worse than BD patients. SC patients also scored significantly lower in sadness (*p<.*01) and disgust recognition (*p<.*05) compared to controls. Moreover, both SC (*p<.*01) and BD patients (*p<.*01) scored significantly lower than controls in fear recognition. This effect remained significant in both groups even though a significant effect of mania (*p<.*01) on fear recognition was observed.

**Table 3:** TASIT.

Emotions	SC (n = 15)	BD (n = 15)	CTR (n = 30)	SC vs. CTR	BD vs. CTR	SC vs. BD
Fear	2.26 (1.16)	2.13 (0.83)	3.5 (0.5)	0.04	0.01	NS
Anger	3.06 (1.09)	3.8 (0.41)	3.96 (0.18)	NS	NS	NS
Sadness	1.46 (1.18)	2.53 (1.18)	3.36 (0.71)	0.0006	NS	NS
Sorpresa	3.53 (0.83)	3.6 (0.63)	3.9 (0.3)	NS	NS	NS
Disgust	1.86 (1.18)	1.93 (0.7)	3.2 (0.8)	0.03	NS	NS
Total score	12.2 (3.38)	14.0 (2.13)	18.0(1.43)	0.00003	0.0003	0.003

Total score and emotions recognition.

In order to explore whether participants performed differently in emotion recognition tasks with low (emotional morphing) and high (TASIT) context processing demands, we compared the total accuracy on both tests. There were significant differences between groups (F(2, 57) = 3.60, *p*<.05). Post-hoc analysis (Tukey HSD, *MS* = 120.99 *df* = 102.09) showed that controls exhibited a better performance in the TASIT than in the emotional morphing (*p*<.05). Conversely, SC (*p* = .95) and BD (*p* = .97) showed a similar performance in both tasks.

In summary, both patient groups showed performance impairment for negative emotions in tasks measuring facial expressions recognition and contextual inference of emotional states. Specifically, both groups had difficulties recognizing fear and sadness expressions. However, SC showed a lower TASIT total score than BD patients. They also exhibited deficits in recognizing anger and disgust expressions, suggesting that this group may have greater emotional processing impairment.

#### Empathy

Descriptive statistics are provided in Table 4. Significant differences between groups (*F*(4,114) = 15.91, *p<.*01) were observed in the situation comprehension accuracy. Post-hoc analysis (Tukey HSD, MS = 328.58, df = 170.27) revealed that SC and BD have a significantly lower comprehension for neutral (both *p<.*01) and accidental (both *p<.*01) situations compared to controls.

**Table 4:** Empathy for pain task. Ratings and reaction times.

		SC (mean±SD)	BD (mean±SD)	CTR (mean±SD)
Comprehension neutral situations (accuracy %)		68.88±34.42	53.33±27.60	90.00±17.83
Comprehension intentional pain situations (accuracy %)		86.66±12.32	90.30±10.57	81.51±9.39
Comprehension accidental pain situations (accuracy %)		38.78±29.84	38.18±21.23	87.27±11.59
Ratings NS	Empathic concern	−1.40±5.84	−2.05±4.71	−2.63±12.05
	Discomfort	−3.74±5.74	−2.85±3.94	−7.82±2.45
	Intention to hurt	−4.23±4.69	−1.44±3.71	−6.90±5.07
	Happiness	−0.22±5.99	2.19±5.48	1.43±4.99
	Correctness	−2.50±5.61	−3.92±3.04	1.55±11.51
	Punishment	−3.74±5.93	−4.12±2.87	−8.14±1.93
Ratings IS	Empathic concern	4.53±3.08	3.71±3.50	1.40±6.43
	Discomfort	5.42±2.46	4.98±2.98	1.73±4.79
	Intention to hurt	5.46±8.34	5.57±2.42	3.46±6.74
	Happiness	−0.24±5.83	2.18±4.97	2.11±3.68
	Correctness	6.26±2.23	6.08±1.95	1.71±5.00
	Punishment	5.40±2.80	5.40±2.85	3.73±4.68
Ratings AS	Empathic concern	2.88±5.21	2.13±4.35	−5.06±2.85
	Discomfort	3.84±3.98	2.21±4.41	−3.24±3.16
	Intention to hurt	1.6±5.73	2.56±4.76	−4.77±3.84
	Happiness	−1.65±5.50	0.40±4.45	−3.66±4.09
	Correctness	3.62±4.58	3.85±4.49	−1.96±2.93
	Punishment	1.73±8.58	2.47±5.30	−4.33±4.01
RTs NS	Comprehension	74271.98±257423.4	10325.12±8963.8	3129.48±1780.1
	Empathic concern	5568.55±3390.00	9558.84±20341.58	2322.25±1164.50
	Discomfort	5028.73±2137.82	8544.84±7241.87	2174.03±768.16
	Intention to hurt	4930.17±1747.83	7894.33±11183.06	2031.96±393.58
	Happiness	5940.93±3455.05	11063.49±10993.72	2870.97±955.37
	Correctness	4810.66±2885.09	9309.48±11993.86	3561.60±1293.92
	Punishment	6405.17±3403.52	4462.17±6473.89	2358.00±406.58
RTs IS	Comprehension	60697.23±202183.8	5118.23±2980.9	2633.66±595.6
	Empathic concern	7184.84±8443.87	6880.25±9949.84	3054.50±1626.37
	Discomfort	6254.80±3539.82	6925.50±9107.57	2424.14±399.07
	Intention to hurt	5973.70±2865.56	5514.33±9831.93	2939.17±539.44
	Happiness	6407.97±3015.21	8841.90±13426.92	3424.86±779.2
	Correctness	5511.21±3247.13	5660.76±2614.83	2614.83±423.58
	Punishment	5702.57±2980.72	6973.84±8392.10	3124.71±320.58
RTs AS	Comprehension	68043.50±241959.6	7611.48±4414.8	2061.32±208.8
	Empathic concern	5090.16±3392.85	5732.36±4731.86	2187.06±1002.91
	Discomfort	5421.59±3452.62	5917.08±5726.28	2745.26±739.92
	Intention to hurt	5201.17±3994.94	4751.88±4248.84	2479.13±313.73
	Happiness	5446.16±3438.22	8872.45±10317.62	2665.37±469.96
	Correctness	4719.03±3083.35	6476.78±6333.69	2930.26±550.81
	Punishment	4379.61±2111.53	5307.60±4805.48	2596.63±357.21

NS =  neutral situations; IS =  intentional situations; AS =  accidental situations; RTs =  reaction times.

Significant differences between groups were found in empathic concern ratings (*F*(4,114) = 88.96, *p<.*05). Post-hoc analysis (Tukey HSD, MS = 43.23, df = 141.90) showed that SC patients rated accidental pain situations higher than controls (*p<.*01). Moreover, differences between groups were observed in discomfort ratings (*F*(4,114) = 21.06, *p<.*05). Post-hoc analysis (Tukey HSD, MS = 14.72, df = 121.52) revealed that both SC (*p<.*01) and BD patients (*p<.*01) had higher ratings than controls for accidental pain situations. Significant differences between groups were also found in ratings of intention to hurt (*F*(4,114) = 2.88, *p<.*05). According to the post-hoc analysis (Tukey HSD, MS = 23.18, df = 152.79), BD patients (*p<.*01) rated accidental situations significantly higher than controls. Finally, differences between groups (*F*(4,114) = 43.95, *p<.*01) were found in punishment ratings. Post-hoc analysis (Tukey HSD, MS = 17.08, df = 124.52) showed that SC rated neutral (*p<.*05) and accidental (*p<.*01) pain situations higher than controls. BD patients also rated accidental pain situations (*p<.*01) higher than controls.

Regarding RTs, significant differences were found in discomfort (*F*(4, 114) = 6.38, *p<.*01). A post-hoc analysis (Tukey HSD, MS = 1642E4, df = 70.69) revealed that BD patients had significantly longer RTs in intentional (*p<.*05) and neutral situations (*p<.*01) than controls. These results remained consistent after covariate analysis (*p<.*01). However, a significant effect of depression on RTs for discomfort in intentional (*p<.*05) and neutral situations (*p<.*05) was observed.

The RTs for intention to hurt also differed between groups (*F*(4,114) = 4.49, *p<.*01). A post-hoc analysis (Tukey HSD, MS = 2195E4, df = 77.74) showed that BD patients had significantly slower RTs in neutral situations (*p<.*01) than controls. Moreover, there were significant differences between groups (*F*(4,114) = 4.75, *p<.*01) in the RTs for correctness. Post-hoc analysis (Tukey HSD, MS = 2316E4, df = 72.58) revealed that BD patients (*p<.*01) showed significantly slower RTs in neutral situations than controls. Although this result was preserved after covariation (*p<.*01), a significant effect of depression (*p<.*05) on RTs for correctness in neutral situations was observed.

Significant differences between groups (*F*(4,114) = 4.62, *p<.*01) were also observed in RTs for punishment. Post-hoc analysis (Tukey HSD, MS = 1320E4, df = 77.69) showed that SC patients (*p<.*05) had slower RTs in neutral situations compared to controls. Furthermore, BD patients (*p<.*05) exhibited slower RTs than controls in intentional situations.

Summarizing, SC and BD patients exhibited difficulties in situation comprehension, suggesting deficits in the ability to distinguish neutral and accidental situations from intentional pain situations regarding empathy for pain in setting with contextual information. Moreover, both patient groups exhibited higher ratings of discomfort and punishment for accidental pain situations. In addition, compared to controls, SC patients presented higher ratings of empathic concern and BD patients showed higher ratings of intention to hurt for accidental pain situations. BD patients exhibited longer RTs in judgments of discomfort, intention to hurt, correctness and punishment. Depressive symptoms had significant effects on delayed RTs in BD patients in discomfort and correctness judgments.

#### Social norms

No significant differences between groups were observed in the break score (*F*(2.57) = 2.04, *p* = .13), nor in the over-adhere score (*F*(2,57) = 1.34, *p* = .26).

### Associations

**Figure 2:**
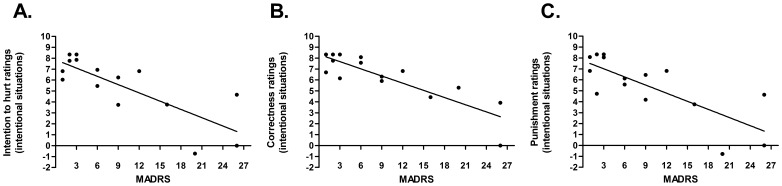
Significant correlations between clinical symptoms and social cognition measures in SC. (A) Negative correlation between depression symptoms and intention to hurt ratings for intentional pain situations. (B) Negative correlation between depression symptoms and correctness ratings for intentional pain situations. (C) Negative correlation between depression symptoms and punishment ratings for intentional pain situations.

Figure 2 summarizes the significant correlations.

#### Correlations between clinical symptoms, executive functions and social cognition measures

In SC, depression levels (MADRS total score) were negatively correlated with intention to hurt (r = −.79), correctness (r = −.86) and punishment (r = −.77) ratings for intentional pain situations.

In BD patients and controls, no significant correlations were found. No correlations between EF and social cognition were identified.

#### Is empathy performance partially explained by emotional processing?

Using the TASIT and the emotional morphing tasks to model performance on emphatic neutral situations evidenced that only the latter has a significant influence.

**Figure 3:**
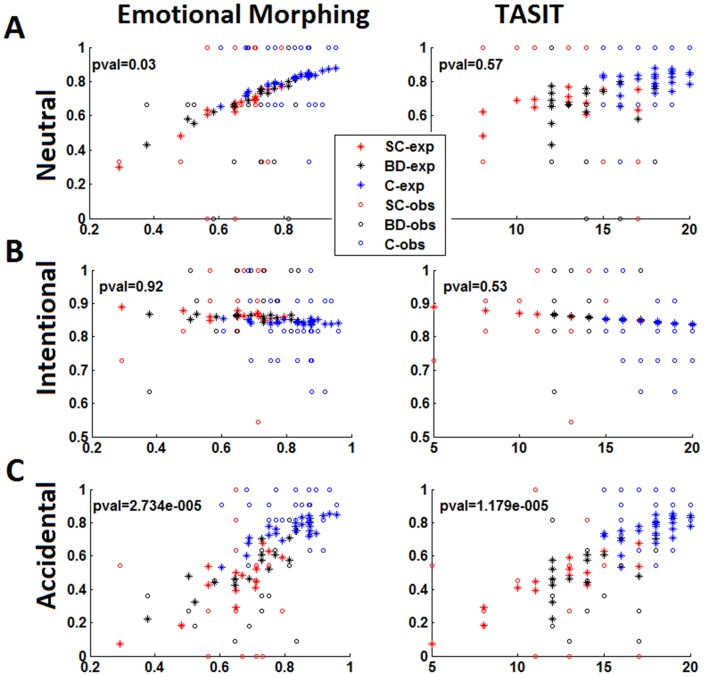
Performance on neutral, intentional and accidental situations during EPT depending on the performance on emotional morphing and TASIT. A) Regression on empathic neutral situations. Observe how the low p-values (5% level of significance) in the emotional morphing evidence a relationship with neutral but no with TASIT. B) Regression on empathic intentional situations. Non significant effects were observed in both (TASIT and emotional morphing) evidencing no dependence among these variables and intentional situations. C) Regression on empathic accidental situations. The performance of accidental situations was depending on both emotional scores. Observe the monotone increasing relationship between both variables and the performance on accidental intention related tasks. Asterisks (*) indicate the expected (exp) values obtained from the data, and circles (o) referred to the observed (obs) measures.

An increment in this variable predicts higher performance on neutral situations (Figure 3A). Conversely, modeling performance in terms of both emotion recognition tasks for intentional conditions yielded no significant effects (Figure 3B).

Finally, performance on both the TASIT and emotional morphing tasks has a significant influence on modeling performance in accidental situations. Increments in both variables predict a better performance on accidental situations. Thus, the global scores for emotional processing tasks predict performance on intentionality attribution on empathy tasks during more ambiguous situations (neutral and accidental). These effects were observed regardless any group differences.

Table 5 summarizes the statistical results.

**Table 5:** Regression scores of emotional variables on empathy performance.

	Value	SD	Wald-statistic	Sig. P-val
**Neutral**				
Intercept	−2.04	0.96	−2.11	0.035
TASIT	0.03	0.06	0.55	0.5773
Emotional Morphing	3.48	1.64	2.12	0.0339
**Intentional**				
Intercept	2.25	0.66	3.41	0.0006
TASIT	−0.02	0.04	−0.62	0.5345
Emotional Morphing	−0.10	1.07	−0.09	0.9260
**Accidental**				
Intercept	−4.35	0.56	−7.76	0.0000
TASIT	0.14	0.03	4.38	0.0000
Emotional Morphing	3.56	0.84	4.19	0.0000

## Discussion

The primary goal of this work was to compare the performance of SC and BD patients on several social cognition domains, assessing the relevance of context processing. We included tasks with different levels of contextual dependence and involvement of real-life scenarios. Emotional morphing provides a more realistic and sensitive measure than static stimuli due to the inclusion of a dynamic method for the presentation of facial expressions. In contrast, the TASIT is a more ecological test of contextual inference of emotional states and requires the integration of cues from face, prosody, gesture and social context to identify the emotions [29], [65]–[67]. We also included an ecological task of empathy for pain in the context of intentional and accidental harms. This task requires the contextual appraisal to infer intentions and subsequently provide empathy responses. Finally, we employed the SNQ, a task that can be solved with social rules learned by explicit knowledge and does not involve social context processing.

Our results showed that both SC and BD patients showed impairments in negative emotion recognition (accentuated in SC with more contextual information present). Moreover, both patient groups exhibited deficits in distinguishing empathy for pain in neutral and accidental situations from intentional situations in settings with contextual information present. In addition, depression levels in SC patients were negatively correlated with performance in empathy. Finally, regardless of group differences, the overall performance on emotion recognition predicted the performance on neutral and accidental situations during the empathy task.

This is the first social cognition study that has compared SC and BD patients in terms of context-sensitive measures of social cognition. Our results suggest that both disorders show deficits in social cognition tasks with greater context sensitivity, while in tasks that can be solved by explicit knowledge they performed normally. Thus, the pattern of social cognition deficits in SC and BD patients may be partially explained by a general impairment of social context processing.

### Deficits in Social Cognition and Contextual Effects

Both patient groups showed more severe impairments for negative emotions in the emotional morphing task. This result was consistent with previous studies [4], [70], [71]. Specifically, both groups had difficulties in recognizing fear expressions. In addition, SC patients showed deficits in disgust and anger recognition, while BD patients had impairments in sadness recognition. This kind of task (dynamic presentation of facial expressions) provides a more realistic and sensitive measure than static stimuli [7], [72], [73] because it closely resembles the moving and dynamic facial stimuli encountered in everyday life situations.

We also included a more ecological task of contextual inference of emotional states (TASIT). In this task, both SC and BD patients performed lower than controls. However, SC exhibited greater deficits than BD patients. These results are supported by previous studies comparing both disorders [27], [28]. These findings are also consistent with previous reports on impaired emotion perception in SC patients [29], [74] on tasks combining facial, gesture, prosodic and contextual information. Our data suggest that although both disorders have deficits in the contextual inference of emotional states, impairments in SC are more severe.

In summary, the results of the emotional processing tasks showed that both SC and BD patients had deficits in recognizing negative emotions. We also found that controls exhibited better performance in the TASIT than in the emotional morphing. However, implicit contextual cues provided in the TASIT did not improve emotion recognition in SC and BD patients. These results are in line with a previous study [65] showing that contextual cues (tone of voice, gesture and dynamic expression) presented in the TASIT normally assist healthy individuals in more accurately identifying emotional expressions [65].

A recent study [75] found intact context processing in SC patients asked to interpret ambiguous facial expressions. The discrepancy between these results and our findings may be explained by the explicit/implicit presentation of contextual information in each study. Lee and colleagues used a paradigm that presented a fear-inducing or surprise-inducing statement immediately followed by image of a face. In this paradigm, contextual cues were explicitly presented by means of a sentence, while our study used a method that requires the implicit appraisal and integration of contextual cues to accurately recognize emotions.

We also employed an ecological measure of empathy for pain in settings with contextual information. In this task, SC and BD patients showed subtle deficits in distinguishing neutral and accidental situations from intentional pain situations. This result is expected since contextual pain cues in accidental and neutral situations are less clear and explicit than in intentional situations. Our data are in line with previous studies in SC [30], [76] and BD patients [6], [32], [77] that have reported deficits in inferring intentionality of others’ actions. These results are also consistent with studies in SC patients [45]–[47] showing social context processing deficits.

Moreover, both patient groups exhibited higher ratings of discomfort and punishment for accidental situations compared to controls. SC patients also presented higher ratings of empathic concern, while BD patients showed higher ratings of intention to hurt for accidental pain situations. Through the contextual appraisal of a situation we can infer intentions of an action and determine the empathizer’s behavioral responses. Thus, these findings may reflect the effects of impairments in SC and BD patients on the contextual appraisal, and the inference of intentionality of pain situations.

Finally, BD patients showed longer RTs in judgments of discomfort, intention to hurt, correctness and punishment. Consistent with these findings, previous studies in BD patients have reported a general decrease in psychomotor speed [78], [79] and longer RTs in empathy tasks [8].

Our results revealed no differences between patient groups and controls on the SNQ. This finding indicates that explicit knowledge of social rules is preserved in both disorders. This is consistent with previous reports showing that SC patients are able to identify violations of social norms [36] and BD patients exhibit appropriate knowledge of social norms [38]. Furthermore, the SNQ can be solved using relatively abstract and universal rules about the world learned by explicit knowledge, and does not measure context processing.

Finally, the logistic regression model suggests that in all groups the emotional processing performance (emotional morphing and TASIT) accurately predicts the ability to infer intentionality in the EPT in more ambiguous scenarios (accidental and neutral situations where differences among patients and controls were observed). In accidental and neutral pain situations, contextual cues are less clear and explicit than in intentional ones, complicating the interpretation of action intentionality. Moreover, the correct appraisal of others’ intentions in situations with subtle contextual clues requires intact recognition ability of emotions. Our findings are supported by previous evidence [3], [29] describing the association between emotional processing and empathy. In addition, the model suggests that the use of basic social skills on emotion recognition is necessary to perform complex abilities such as empathy evaluation.

To summarize, SC and BD patients showed impairments in emotional processing and empathy tasks. However, empathy deficits seem to be more subtle and dependent on emotional processing. The pattern of performance of both patient groups suggests that SC and BD patients exhibit deficits in those social cognition tasks with greater context sensitivity and involvement of real-life scenarios (TASIT and EPT). In the SNQ, an explicit knowledge task, performance of both patient groups mirrored controls. These findings are consistent with previous reports of social contexts processing deficits in SC patients [45]–[47]. Moreover, our data provide preliminary evidence of impairments in social context processing in BD patients. Our results also indicate that deficits in emotional processing tasks using context cues are less severe in BD than in SC patients.

### The Relationship between Clinical Symptoms, Executive Functions and Social Cognition Measures

Our results showed negative correlations between depression levels and empathy task performance in SC patients. These results are consistent with previous studies in SC [80], [81] that report an association between depressive symptoms severity and social functioning. Furthermore, in the EPT, although significant differences in RTs were preserved after covariation, we found that depressive symptoms had significant covariant effects on delayed RTs of BD patients in discomfort and correctness judgments. These findings are supported by previous studies in BD patients [82], [83] showing that depressive symptoms were associated with dysfunction in psychomotor speed.

Regarding EF, our results showed that both patient groups performed worse than controls, but SC exhibited greater deficits than BD patients. Nevertheless, the social cognitive performance was not associated with EF. This may be explained by the high threshold (.0008) used to adjust the α level for multiple correlations. Although the IFS is a useful instrument to detect executive dysfunction in diseases involving the prefrontal cortex [61], [62], it cannot serve as the sole tool to assess EF. Future studies should employ a more exhaustive EF evaluation to explore the relationship between executive functioning and contextual social cognition.

### Limitations and Future Directions

Some important limitations of this study should be acknowledged. First, every participant was taking psychoactive drugs at the time of this study, which can influence cognitive functioning. We cannot rule out the possibility that medications influenced the context processing and social cognition performance. The sample size was relatively small, and therefore more subtle differences may have been missed due to a lack of statistical power and multiple comparison adjustments. However, our sample size is enough for the type of analyses performed here [84]–[86] and is similar to previous studies in neuropsychiatric populations [82], [87]–[89]. Further studies should assess the effect of context processing in social cognition domains in larger samples of SC and BD patients.

Moreover, not all BD patients were euthymic. A more homogenous sample could modify the results of social cognition tasks that are strongly modulated by emotional state. In addition, this study included BD II patients, possibly indicating that impairments in social cognition found in this study may not be exclusively related to psychotic forms of these disorders. Future investigations should take into account the subtypes of SC and BD. Larger subgroups of BD patients in euthymic, manic, hypomanic or depressed states should also be assessed.

Finally, in this study social norms knowledge was assessed by means of a self-report questionnaire that implies lower complexity than the other social cognition tasks. Further studies should evaluate social norms knowledge using more ecological measures with different levels of context processing requirements.

### Conclusions

Our study documents social contexts processing impairments in SC patients and BD patients. The results showed that both patient groups exhibit deficits in ecological measures of social cognition with greater context sensitivity (TASIT and EPT), while in the SNQ, a task that can be solved by explicit knowledge, they performed normally. These deficits would be related to a general impairment in the capacity to implicitly integrate contextual cues.

SC and BD partially share genetic susceptibility [49], [50] and symptomatology [54]; and also have similar ages of onset, sex distributions, and prevalence [51]. Despite these similarities, there are relatively few social cognition studies comparing both disorders. To our knowledge, this is the first study that assesses the effects of contextual social cognition performance comparing SC and BD patients.

From a theoretical perspective, our results support a recently proposed social context network model (SCNM) [39] that describes the contextual influence on social cognitive processing as dependent on a frontotemporal network which: 1) updates contextual cues and uses them to make predictions (frontal areas), and 2) consolidates context-social target associative learning (temporal regions). At the structural and functional level, the most affected brain areas in SC are the temporal and frontal areas [90], [91]. Following this model, our results suggests that the pattern of social cognition deficits in SC may be partially explained by a general impairment of social context processing which is the result of an abnormal fronto-temporal network.

Volumetric reductions and functional impairments are also present in prefrontal regions in BD, while involvement of temporal lobe structures seems to be a feature more common in SC [92]–[95]. These differences in the degree of disruption in fronto-temporal structures may explain the more severe social context processing impairments observed in SC patients. Although our findings provide preliminary evidence supporting this hypothesis, future studies in neuropsychiatric populations should strictly control for the context dependency levels of social cognition tasks, including measures with context processing requirements and context-free tests or manipulation of experimentally contextual cues.

Several reports in SC [96]–[98] show a general deficit in contextual processing of nonsocial information. Furthermore, it has been suggested [74] that in individuals with SC, better processing of nonsocial contexts is associated with stronger influence of context on emotional processing tasks. Thus, it is possible that in SC patients, similar mechanisms may influence the contextual processing of both social and non-social information. Therefore, in SC, frontotemporal networks would also be involved in the processing of anticipatory predictions based on context update (frontal areas) and the learning of context-target associations (temporal regions) for non-social information. In BD, further studies should assess the relationship between context processing of social and nonsocial information.

From a clinical perspective, our findings may have important implications for the non-pharmacological treatment of SC and BD patients. Although social cognition deficits are not considered core symptoms of SC or BD, these should be taken into account for the patients’ cognitive evaluation and rehabilitation. In addition, our results suggest that ecological measures with context processing requirements are sensitive tools that should be applied in the social cognition assessment of these patients. Moreover, incorporating naturalistic environments into treatment may help SC and BD adults to generalize learned social skills. Despite the challenge of implementation, intervention programs, which would teach implicit rules for interpreting unpredictable social contexts, should be put into place. Learning to appraise contextual cues may improve social skills of SC and BD patients.

## Supporting Information

File S1 Generalized Linear Model Details. A description of the GLM procedure.
(DOC)

File S2 Executive Functions Assessment. A detailed comparison of performance on the eight IFS subtests.
(DOC)
