# Supplementary material for: Contextual Social Cognition Impairments in Schizophrenia and Bipolar Disorder
Source: PLoS One. 2013 Mar 8;8(3):e57664. doi: 10.1371/journal.pone.0057664 (PMC3592887; doi:10.1371/journal.pone.0057664)
Supplement: File S1 — Generalized Linear Model Details. A description of the GLM procedure. (DOC) [file pone.0057664.s001.doc]

**S1 Generalized Linear Model Details**

A realization of a binomial variable Y ~ B(n,p), states how many times out of n an event with probability of occurrence p succeeded. In an experimental environment, a participant is presented with n trials, and individual answers are then classified into binomial format (correct, incorrect). As a result, we can create a model using a binomial outcome. However, the probability of answering properly depends on certain features and can vary among individuals. To account for this variability, the probability of success for each participant could be parameterized on other variables. The binomial logit framework allows for this feature, since it models the outcome of the test for each individual:

Y­­­_i_ ~ B(n,p_i_), where p_i_ = logit (X_i_ β),

Where X_i_ is a set of independent variables and β is the estimated parameter. The function logit maps real numbers to probabilities. The maximum likelihood estimation is achieved through Fisher’s method of scoring. The asymptotic properties of the estimator allow for significance tests on the parameters, calculating which parameter has an important influence on the response. The influence of the parameters on the response can be interpreted in terms of the odds of success, i.e.

p_i_/(1-p_i_) = exp(X_i_β) = exp(X_i0_β_i0_+ X_i1_β_i1_+…+ X_iP_β_iP_)

= exp(X_i0_β_i0_) exp(X_i1_β_i1_)…exp(X_iP_β_iP_)

An increment of 1 unit in the parameter X_i0_ implies :

p_i_/(1-p_i_) = exp[(X_i0_+1)β_i0_] exp(X_i1_β_i1_)…exp(X_iP_β_iP_)

= exp(β_i0_) exp(X_i0_ β_i0_) exp(X_i1_β_i1_)…exp(X_iP_β_iP_)

A multiplication of the odds of success by a factor exp(β_i0_). Thus, if β_i0_>0 then the bigger X_i0_, the greater the odds and consequently, the probability of success. If β_i0_<0, then the bigger X_i0_, the lower the odds and consequently, the probability of success. This analysis can be performed with every parameter to analyze its influence on the probability of correctly answering a question.
