# Supplementary material for: Contextual Social Cognition Impairments in Schizophrenia and Bipolar Disorder
Source: PLoS One. 2013 Mar 8;8(3):e57664. doi: 10.1371/journal.pone.0057664 (PMC3592887; doi:10.1371/journal.pone.0057664)
Supplement: File S2 — Executive Functions Assessment. A detailed comparison of performance on the eight IFS subtests. (DOC) [file pone.0057664.s002.doc]

**S2 Executive Functions Assessment Results**

A detailed comparison of the performance on each of the eight IFS subtests indicated that the groups differed significantly (*F*(2,57) = 12.67, *p <* .01) on the conflictive instructions subscale. The post-hoc analysis (Tukey HSD, MS = .63 df = 57) demonstrated that SC patients had lower scores than BD patients (*p <*.01) and controls (*p <*.01). We also observed group differences (*F*(2,57) = 9.00, *p <*.01) in motor inhibitory control. Post-hoc comparisons (Tukey HSD, MS = .71, df = 57) showed that SC performed worse than controls (*p <*.01). Furthermore, differences between groups (*F*(2,57) = 9.95, *p <*.01) were observed on the backward digits span. The post-hoc analysis revealed (Tukey HSD, MS = 1.95, df = 57) lower scores in SC patients (*p <*.01) and BD patients (*p <*.01) compared with controls. In addition, the groups differed significantly (*F*(2,57) = 12.67, *p <*.01) in verbal working memory. Post-hoc analysis (Tukey HSD, MS = .50, df = 57) showed that SC patients had lower scores than BD patients (*p <*.01) and controls (*p <*.01). Significant differences (*F*(2,57) = 15.63, *p <*.01) were also observed in the abstraction capacity. Post-hoc comparisons (Tukey HSD, MS = 15.47, df = 57.00) evidenced that SC patients performed worse (*p <*.01) than controls. BD patients also performed worse than controls (*p <*.05), but significantly better than SC patients (*p <*.05). Finally, differences between groups (*F*(2,57) = 9.71, *p <*.01) were observed in verbal inhibitory control. Post-hoc analysis (Tukey HSD, MS = 2.12, df = 57.00) revealed that SC patients had a lower performance than BD patients (*p <*.05) and controls (*p <*.01).
